# Gene/Environment Interaction in Atherosclerosis: An Example of Clinical Medicine as Seen from the Evolutionary Perspective

**DOI:** 10.4061/2010/654078

**Published:** 2010-03-25

**Authors:** Gerhard Mertens

**Affiliations:** Department of Clinical Biology, Antwerp University Hospital, University of Antwerp, Wilrijkstraat 10, 2650 Edegem, Belgium

## Abstract

Evolutionary medicine is the application of evolution theory to understanding health and disease. It provides a complementary scientific approach to the present mechanistic explanations that dominate medical science, and particularly medical education. The chronic multifactorial disease of atherosclerosis clearly illustrates the Darwinian paradigm. Recent research, combining the effects of genes and environment, has provided surprising clues to the pathogenesis of this major public health problem. This example makes a strong case for recognizing evolution biology as a basic science for medicine.

## 1. Introduction

“Nothing in Biology Makes Sense Except in the Light of Evolution” is a 1973 essay by the great evolutionary biologist Dobzhansky [1]. He writes: “Seen in the light of evolution, biology is—perhaps—intellectually the most satisfying and inspiring science. But without this light, it becomes a pile of sundry facts, some of them interesting or curious but making no meaningful picture as a whole.”

Evolution's role is also of paramount importance in the sub-discipline of biology that addresses health and disease in humans. 

The co-evolution of man and pathogenic microorganisms, as well as man's persistent vulnerability to chronic diseases should all be viewed in the context of continuing evolution. These subjects form the core of “evolutionary medicine,” also known as “Darwinian medicine” [2]. 

Evolutionary medicine is not a form of “alternative medicine.” Actually, it is the opposite, complementing scientific evidence-based medicine by not only seeking the immediate causes of disease—the “how”—but also the longer-term reasons for the existence of these diseases—the “why.”

While the practice of medicine by itself aims at counteracting natural selection, the following pledge for evolutionary medicine is not paradoxical. Indeed, it is by improving the knowledge on evolution biology of clinicians, learning them to see health and disease from an evolutionary perspective, that more effective treatment and preventive strategies can be developed.

Atherosclerosis is an example of the contribution of Darwinian insights into the aetiology of a major chronic disease. Recent research, combining the effects of genes and environment, has provided new clues to the cause of this leading cause of global morbidity and mortality.

## 2. Genetic Factors

### 2.1. Alleles of the Apo E Gene

Genetic factors have an unquestionable role in atherosclerosis [3].

The apo E gene [4] is located at chromosome 19q13.2. Among the variants of this gene, the alleles epsilon2, epsilon3 and epsilon4 constitute the common polymorphism found in most populations, the epsilon3 allele being the most frequent (>0.60) in all populations studied. The polymorphism however is not neutral but has functional effects on lipoprotein metabolism, mediated through the hepatic binding, uptake, and catabolism of chylomicrons, chylomicron remnants, very low density lipoproteins and high density lipoproteins. Epidemiological studies have demonstrated that the primary genetic risk factor predisposing to atherosclerosis is the epsilon4 allele of the apo E gene [5].

### 2.2. Population Genetics and Evolution

The apo E epsilon3 allele is the most frequent in all studied human populations, its frequency being highest (0.85–0.90) among the populations of the Mediterranean basin. Correspondingly, the apo E epsilon4 allele has the lowest frequencies (0.05–0.13) in Mediterranean populations, who were also the first to establish agricultural economy. On the other hand, the frequency of the epsilon4 allele remains higher in populations like Pygmies (0.41), Khoi San (0.37), aborigines of Malaysia (0.24) and Australia (0.26), Papuans (0.37), some Native Americans (0.28) and Lapps (0.31), where an economy of foraging still exists or food supply is (or was until the recent past) scarce and sporadically available [6].

From the evolutionary perspective, these observations lead to the conclusion that the epsilon4 allele must be the ancestral allele [7]. As an evolutionary relic from the pre-agricultural history of *Homo sapiens*, it is not adapted to a modern, highly nutrient-rich culture [8]. Through the process of natural selection, other, better adapted alleles are replacing the epsilon4 allele, generation after generation. Still, the allele has at present, 10 000 years after the discovery of agriculture, still a high frequency. The mechanism of natural selection eliminating the epsilon4 allele after the introduction of agriculture, is supported by the fact that in contemporary populations with a longer tradition of agriculture the allele is less frequent than in populations that have shifted more recently to agriculture, and even much less frequent than in “primitive” populations with a hunter-and-gatherer lifestyle today [9].

## 3. Environmental Factors

### 3.1. Multiple Factors

Aside from genetic factors, there are also environmental—other than dietary—factors to consider in atherosclerosis.

It is widely known that cigarette smoking constitutes a major environmental risk factor for atherosclerosis. Gene/environment interaction has also been demonstrated in this regard. Indeed, a synergistic effect between cigarette smoking and carrier state of the apo E epsilon4 allele increases the risk of atherosclerosis to a large extent [10].

### 3.2. Role of Chlamydia Pneumoniae

An environmental factor of recent interest in the pathogenesis of atherosclerosis is bacterial infection.

First, histopathological studies in humans have shown—contrasting with what was traditionally assumed—that triglycerides and cholesterol do not simply accumulate on the inner surface of the artery, but within the arterial wall itself. Within atherosclerotic lesions—somewhat surprisingly—bacteria have been demonstrated, especially *Chlamydia pneumoniae* [11]. This is an intracellular pathogen, known as a leading cause of human respiratory tract infections worldwide. In order to play a causative role in chronic disease, *C. pneumoniae* would need to persist within infected tissue for extended periods of time, thereby stimulating a chronic inflammatory response. *C. pneumoniae* has been shown to disseminate systemically from the lungs through infected peripheral blood mononuclear cells and to localize in arteries where it may infect endothelial cells, vascular smooth muscle cells, monocytes/macrophages and promote an inflammatory atherogenous process [12]. 

Also using an animal model, *C. pneumoniae* have been demonstrated in atherosclerotic lesions but not in normal arteries [13]. Apo E knockout mice, which spontaneously develop atherosclerosis, and C57BL/6J mice, which only develop atherosclerosis on an atherogenic diet, were evaluated. Following intranasal inoculations of apo E knockout mice, *C. pneumoniae* were detected in lung, aorta, and spleen for 20 weeks in 25 to 100% of mice. In the aorta, *C. pneumoniae* were detected within the atherosclerotic lesion. In C57BL/6J mice on a nonatherogenic diet, *C. pneumoniae* were detected in the aorta within 2 weeks after intranasal inoculation. The persistence of *C. pneumoniae * in atheromas suggests a tropism of *C. pneumoniae* to the lesion. These mouse models should be useful for studying the pathogenic role of *C. pneumoniae* in atherosclerosis.

The synergistic effect between the genetic and the environmental risk factors has also been elucidated [14]. The gene product of the epsilon4 allele of the apo E gene functions as a receptor protein permitting *C. pneumoniae* to penetrate the endothelial cells of the arterial wall. Thus, all pieces of the puzzle come together and we get close to proof that *C. pneumoniae* is causally involved in atherosclerosis [15].

### 3.3. Resolving the “Passive Smoker's Paradox”

When a respiratory infection is viewed as the primary cause of atherosclerosis, the paradox of “passive smoking” [16] is resolved. Traditionally, the risk of atherosclerosis by passive smoking is attributed to the direct effect of toxic compounds in the smoke. Still, the link between second-hand exposure to smoke and the increased atherosclerosis risk appears to be quantitatively problematic. Compared with a nonsmoker, a heavy smoker increases his risk by 100%. Living with a smoker increases the risk by 30%, although the exposure to smoke is 100 times smaller in the passive smoker [17]. Thus, the relative effect of passive smoking seems out of proportion to the small amount of smoke inhaled. This paradox goes up in smoke if the primary cause of atherosclerosis is infection, spread by airway transmission. Smoking indeed increases the susceptibility to respiratory infection and living together with a smoker increases the risk of acquiring the same infection. 

The insight that a frequent and potentially mortal disease could be caused by a bacterium opens new doors for treatment and prevention.

## 4. Synthesis and Conclusion

The consideration of apo E alleles and smoking provides two illustrations of a comprehensive approach to the causation of atherosclerosis. Instead of accumulating risk factors, this approach seeks a unified theory of causation. Infection as a primary cause of atherosclerosis provides a conceptually cohesive framework for understanding the role of noninfectious environmental risk factors, such as smoking and diet. 

The example of atherosclerosis witnesses the progress made at the intersection of medicine and evolution, of both humans and pathogenic microorganisms. The involvement of *C. pneumoniae* in atherosclerosis has been shown by seroepidemiological and pathological studies, in vivo and in vitro studies and in clinical antibiotic treatment trials. This factor can be identified, modified and may be a therapeutic target. 

Prospective interventionist studies on humans to prove the relation between genetic evolution and an environmental risk factor are difficult because of ethical and—above all—practical reasons (long generation time, limited number of offspring). Therefore, continued basic research on apo E knockout mice as well as C57BL/6J mice is needed for the future [18].

The key questions in evolutionary medicine are centred around which aspects of modern environment and lifestyle are pathogenic. That is what we ultimately need to know and it should be clear to all: Medicine Needs Evolution [19].
